# An ipRGC-influenced/Non-Visual Spectral Occupant Model (iNSOM) for lighting design, Part 1: Light simulation method

**DOI:** 10.1177/14771535251368379

**Published:** 2025-10-09

**Authors:** A Alight, JA Jakubiec

**Affiliations:** a John H. Daniels Faculty of Architecture, Landscape and Design, University of Toronto, Toronto, ON, Canada; b School of the Environment, University of Toronto, Toronto, ON, Canada

## Abstract

This paper describes the light simulation development of an intrinsically photosensitive retinal ganglion cell (ipRGC)-influenced/Non-Visual Spectral Occupant Model (iNSOM). The light simulation framework of iNSOM combines multi-spectral lighting simulations into time-series annualized melanopic irradiance. The model integrates daylight, electric light and light from screen devices into the calculation methods. The applicability of the light simulation method is demonstrated using an example hospital ward model and tested under three daylight, electric light and evening monitor screen use scenarios.

## 1. Introduction

Circadian rhythms are a key biological regulator that influence our metabolism, behaviour and hormone levels. The word circadian means about a day; however, light exposure is required to entrain the circadian system, synchronizing it to the actual length of a day.^1^ The response to light which entrains our circadian system is primarily mediated by the response of a photopigment within intrinsically photosensitive retinal ganglion cells (ipRGCs) located throughout the retina called melanopsin.^2,3^ Melanopsin is sensitive to shorter wavelengths of light (peak 480 nm) than the photopic visual system (peak 555 nm).^4,5^ ipRGC-influenced responses to light can advance or delay circadian rhythms depending on the time of exposure throughout the day, intensity and duration.^6–9^

This paper describes a pilot method for constructing high-resolution spectral irradiance light simulations using Radiance^10^ (with sky models from ALFA^11^), melanopic irradiance conversions and time-series annual lighting calculations. We develop an annual workflow for the creation of time-series spectral irradiance due to dynamic signals of daylighting, electric lighting and light from self-luminous device screens. Then we evaluate the light simulations using a series of different lighting design strategies involving both daylight and electric light exposure. The method proposed for the generation of annual melanopic irradiance from multiple light sources consists of the first half of a two-part modelling system called iNSOM (ipRGC-Influenced/Non-Visual Spectral Occupant Model). A follow-up paper considers how typical predicted melanopic irradiance patterns throughout the year can be used to predict non-visual effects on human beings. The goal of this paper is to introduce a method to annually simulate spectrally specific irradiance from electric luminaires, screen devices and daylight that are converted to α-opic irradiances for assessing ipRGC-influenced light responses.

## 2. Previous work

### 2.1 Light simulation methods

For over a decade, lighting simulation tools and methods have begun to address the spectrally specific qualities of lighting in spaces. Geisler-Moroder and Dür^12^ created a modified version of Radiance^10^ to predict 81 channels (5 nm interval) of spectral irradiance; however, it did not extend to coloured sky models and was never publicly released. Geisler-Moroder and Dür^13^ also found that the spectral composition of light simulated in spaces with many interreflections were more accurate and distinct when calculating interreflections using more than three colour channels.^14^ Mardaljevic *et al*. developed a lighting simulation tool based on Radiance using a daylight coefficient approach,^15,16^ tracking light exposure from sun and diffuse sky sources. The spectra of received light is resolved according to CIE standard daylight illuminants^17^ for solar beam radiation (D55), overcast sky diffuse radiation (D65) and clear sky diffuse radiation (D75). The Mardaljevic *et al*. method accounts for spectral information due to the sky source but assumes spectrally neutral transmission and interreflections.

Lark implements a nine-channel simulation methodology, utilizing three separate three-channel Radiance calculations. Lark simulations use spatially uniform sky spectra (but varying luminance) and spectrally specific material properties. Users of Lark define the sky spectrum for each simulation as a separate input which is applied as a uniform spectral power distribution on top of a CIE standard sky model.^18,19^ Lark also supports a simplified three-channel implementation with different weights than standard Radiance RGB calculations for computational speed. Pierson *et al*.^20^ and Balakrishnan and Jakubiec^21^ implemented the Perez sky luminance distribution as an extension within Lark, still with a uniform spectrum. An addition to Lark, OWL^22^ automates the selection of sky spectral power distributions and allocates them spatially across a 145-patch sky dome within Lark simulations based on the work of Diakite-Kortlever and Knoop.^23^ Recently, Lark 2.0 was released.^24^ Lark 2.0 can perform time-series simulations under the assumption of a constant CIE D65 diffuse sky spectral power distribution with luminance varying according to the Perez sky model^25^ and equal-energy suns. Lark 2.0 also added the ability to simulate luminaires with known lamp spectral power distributions.

Konis^26^ used the three-channel implementation of Lark’s original version to compute climate-based spectral irradiance calculations using a custom tool known as the circadian design assist tool (CDAT). He chose sky spectra based on cloud cover data from a climate file between the following: a Correlated Colour Temperature (CCT) = 25 000 K sky (clear sky conditions), a CCT = 7000 K sky (10% to 50% cloud cover) and a CCT = 5000 K sky (60% cloud cover to overcast). In CDAT, sky dome luminance distributions are based on the Perez all-weather sky.^25^ Simulations are run individually for each hour in the climate file or a user-specified subset of the climate year.

ALFA implements a Radiance-based method for 81-channel spectral calculations at 5 nm wavelength intervals between 380 nm and 780 nm and can account for light from the sun, sky and luminaires.^11^ Utilizing many spectral calculation bins may increase the tool’s sensitivity to the spectra of interior material reflectance and glazing transmittance.^27^ In addition, more spectral bandwidth increases the ability to assess sources with narrow peaks such as fluorescent lamps or blue LEDs. ALFA uses idealized sky models based on physics-based calculations performed with the libRadtran^28^ atmospheric radiative transfer library based on atmospheric molecular and aerosol profiles measured by the Air Force Geophysics Laboratory,^29,30^ variable ground albedo^31^ and an optional dense cloud layer. LibRadtran-based skies have been shown to be highly accurate when compared to measured data,^32^ and ALFA validations have shown that scaling simulation results according to mean global horizontal irradiance produces simulated spectra with no bias compared to measured data.^19^

Recently Radiance 6 ^33^ was released, which implements multi-channel spectral simulation capacities natively up to a default maximum of 24 evenly spaced spectral bins. Alongside this expansion of the calculation engine, Bruneton and Neyret’s^34^ physics-based spectral sky model was implemented in the tool. Their sky model’s luminance distribution has been favourably compared to the CIE ideal sky model number 12 (standard, low turbidity) although the spectral model suffers from overestimation of light from the horizon.

With the exception of Mardaljevic *et al*.,^14^ Lark 2.0^24^ and Radiance 6,^33^ existing ipRGC-influenced light calculations are developed around point-in-time simulations. Climate-based workflows that account for changing sky spectra are largely not possible without running many individual simulations with different sky conditions.^35,36^ Although the Mardaljevic *et al*.,^14^ Lark 2.0^24^ and CDAT^26^ methods offer annual lighting analysis potential, they lose spectral accuracy by reducing the number of spectral channels and using uniform sky colour distributions. Besides Lark 2.0, they also cannot account for electric light sources. As noted in the description of each spectral simulation method, the determination of sky spectral distribution is another point of inconsistency among existing spectral calculation tools. Mardaljevic *et al*.^14^ used CIE standard illuminant spectra, Lark’s original version requires manual input of spectra, Lark 2.0 uses somewhat different CIE standard illuminant spectra and equal-energy suns and CDAT chooses between three pre-defined options based upon CCT. ALFA and Radiance 6’s implementation of the Bruneton and Neyret sky model determine a spatially varying sky spectrum within the simulation based on physical atmospheric processes but without use of climate-specific data included in weather files. None of these methods have been directly validated against measured sky spectral data.

### 2.2 Electric lighting

Although daylight outperforms electric light in circadian efficacy,^37^ the ability to incorporate electric lighting into an evaluative model is important because most buildings have electric lighting. Electric lighting is necessary when daylight is insufficient, and it is the primary source of potentially disruptive light at night. Indeed, the WELL Standard suggests that equivalent melanopic lux, EML > 150 lx (melanopic equivalent daylight illuminance, $\;E_{v,\;\mathrm{mel}}^{D65}>136\;\mathrm{lx}$ , or $E_{e,\mathrm{mel}}>0.18\;W\;m^{2}$ ) threshold must be able to be met with electric lighting alone.^38^ The use of self-luminous screens is also a prevalent artificial light source that is important to account for which can have direct biological effects on people.^39^ Electric lighting can be beneficial when daylight is not sufficient, and it can be harmful when a light stimulus is provided at biologically disruptive times. Most current ipRGC-influenced light evaluation methods and metrics besides WELL and Brown *et al*.^40^– which will be summarized in the follow-up to this paper – are based upon daylighting; therefore, they cannot assess the phase shifting and melatonin suppressing effects of electric lighting at night.^26,37,41,42^ The differing effects of light exposure during the night versus during the day results in a limitation to most threshold-based evaluation criteria that do not consider the time of light exposure.^14,43^ Brennan and Collins^44^ developed a framework to simulate daylight and electric light with fixed spectral assumptions of CCT = 4000 K or CCT = 6500 K using Daysim and Radiance. However, their framework approach is limited by spectral inaccuracy as Daysim is a single-channel, spectrally neutral simulation tool.^45^ Lark 2.0, ALFA and Radiance 6 are all able to support the simulation of electric lighting^10,24^ at their native spectral bandwidths.

## 3. Method

### 3.1 Geometric model and occupant locations

**Figure 1:**
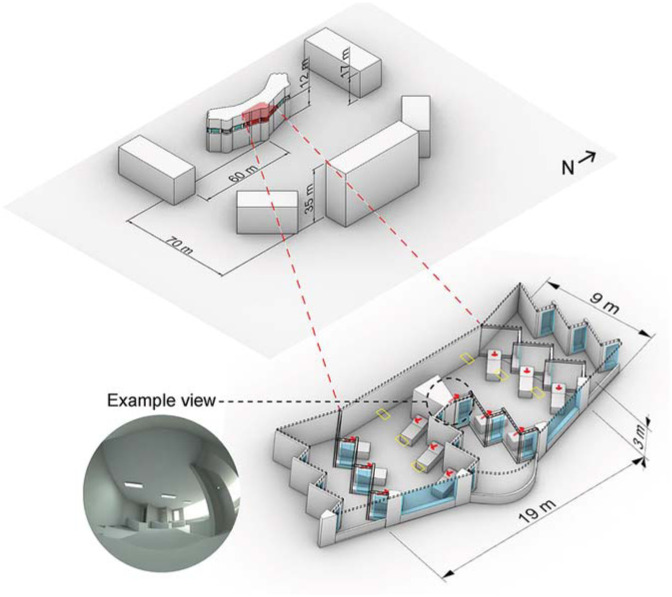
Shared hospital ward model and simulated view directions

We use a model of a daylit ward from the Ng Teng Fong hospital^46^ as an example for lighting analysis, which is depicted in Figure 1. This building was chosen as a realistic and well-daylit architecture where occupants are stationary for long periods of time. The model faces east and has a large potential to receive daylight, especially during the morning. There exists a moderate urban context around the simulation model with buildings between 17 m and 35 m tall. The simulated room itself is 12 m above grade. Twelve views are modelled at the head of each patient bed, reclined at a 30° angle, which are indicated by red arrows in Figure 1 and illustrated with a rendering of an example view. This fixed view does neither encompass all aspects of behaviour such as looking around, changing positions, nor movement throughout a space or between spaces. We suggest that it is useful to judge the impacts of lighting – daylight, shading, electric lighting and controls – on the most typical positions and views within the space.

### 3.2 Material properties

**Figure 2:**
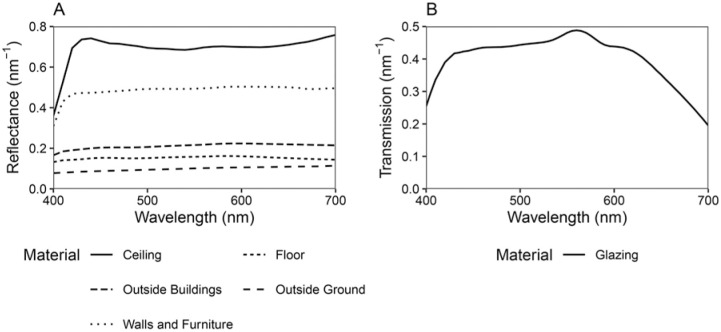
(a) Material spectral reflectance and (b) glazing spectral transmittance

We chose a neutral material palette with melanopic/photopic (M/P) reflectance ratios close to one. Glazing transmittance data was also selected to have an M/P ratio close to one as determined using the LBNL Optics software tool.^47^ Previous work by the authors explored the photobiological effects differences with diverse spectral coloured material reflectance and transmittance.^27^ Summary reflectance and transmittance properties of the materials can be found in Table 1, and spectral reflectance and transmittance data is shown in Figure 2.

**Table 1:** Materials and glazing properties

Surface type	Visible reflectance or transmittance (%)	Melanopic reflectance or transmittance (%)	M/P ratio
Wall and furniture	49.6	48.7	0.98
Ceiling	69.6	70.4	1.01
Floor	15.8	15.3	0.97
Outside buildings	21.7	20.6	0.95
Outside ground	10.1	9.3	0.92
Glazing	45.3	46.0	1.01

### 3.3 Spectral irradiance calculations

Spectral irradiance calculations were completed using Radiance 6 ^33^ using 20, 20 nm spectral bins (ranging from 380 nm to 780 nm) and with ALFA’s^11^ sky spectral distribution models based on solar position and a ‘hazy’ atmospheric profile.^19^ In this study, we used Radiance 6 as the simulation engine with 65,536 primary ray samples per view and 6 ambient bounces to minimize simulation-based uncertainty.

Spectral irradiances are converted into α-opic irradiances using the methodology published by the CIE for assessing ipRGC-influenced light responses.^48^ Although EML is not a standard quantity, to situate our values with older research we note that melanopic irradiance of 1 W m^−2^ is equivalent to melanopic illuminance (EML) of 832 lx. For reference, *E*_e,mel_ of 1 W m^−2^ is equivalent to melanopic equivalent daylight (D65) illuminance, $E_{v,\mathrm{mel}}^{D65}$ of 754 lx.

### 3.4 Electric light sources

**Figure 3:**
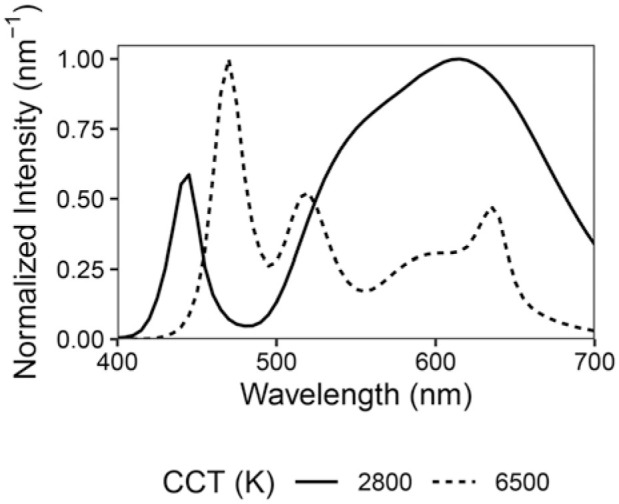
LED lighting spectral power distributions normalized about their peaks

Electric lights can be controlled in terms of their intensity and spectral power distribution, such as by using daylight dimming systems or solid state LED lighting systems. Accordingly, iNSOM’s electric light calculation aims to achieve plausible annual results as well as the ability to adjust for realistic electric light exposures due to luminaires and screens. This enables the assessment of control systems and behaviour on exposure to light and resulting photobiological effects. Radiance 6 is used to calculate spectral irradiance from luminaires using an IES file representing a diffused ceiling-mounted linear direct luminaire. Six luminaires, three on each side of the hospital ward, were located equidistant between the foot of the beds in Figure 1 and are identified by the yellow rectangles in the figure. Two LED spectra are utilized for annual simulations identified by a CCT of 6500 K and 2800 K which are illustrated in Figure 3. These were chosen to represent a ‘daylight’ blue-enriched spectra as well as a warmer colour temperature lamp that could plausibly be used during the evening to reduce ipRGC-influenced biological effects. Each luminaire emits luminous intensity = 3900 lm in a well-diffused distribution. This setup provides on average ~300 photopic lux of ambient electric light to a virtual workplane in the virtual space. We do not simulate other luminaire types such as examination lights. The 6500 K spectrum has a 1.36 M/P ratio, while the 2800 K spectrum’s M/P ratio is 0.38. In simulating both cases, luminous flux defined in the IES file is held constant by scaling the radiance flux proportional to the spectral power distributions shown in Figure 3.

**Figure 4:**
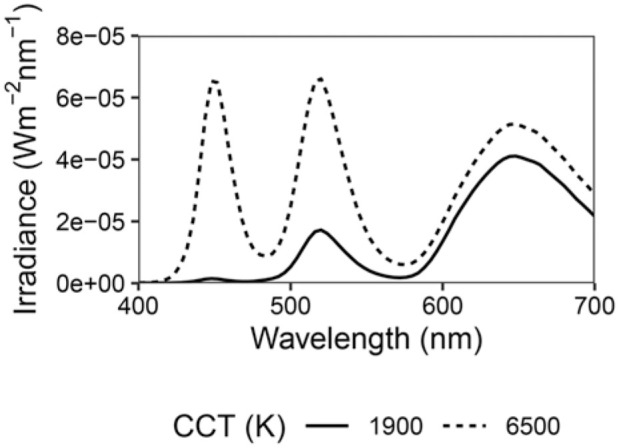
Spectral irradiance received at the eye from an electronic screen at two colour temperature settings

We also predict the effects of using blue-enriched monitor or tablet screens during the entire day versus using a warm colour shift to avoid ipRGC-influenced effects such as melatonin suppression and circadian phase delay in the evening. Measurements from the website f.luxometer^49^ were selected for this purpose of 6500 K (*E*_e,mel_ = 0.113 W m^−2^, $E_{v,\mathrm{mel}}^{D65}$ = 85.2 lx) and 1900 K CCT (*E*_e,mel_ = 0.0183 W m^−2^, $E_{v,\mathrm{mel}}^{D65}$ = 13.8 lx), which represent a bright LED screen at a typical viewing distance and are depicted in Figure 4 showing spectral irradiance received at the eye. While light exposure from screen devices will vary by device, screen type, settings, viewing distance and viewing angle, these represent reasonable light exposures based on the author’s experience.

### 3.5 Annual daylight simulation method

**Figure 5:**
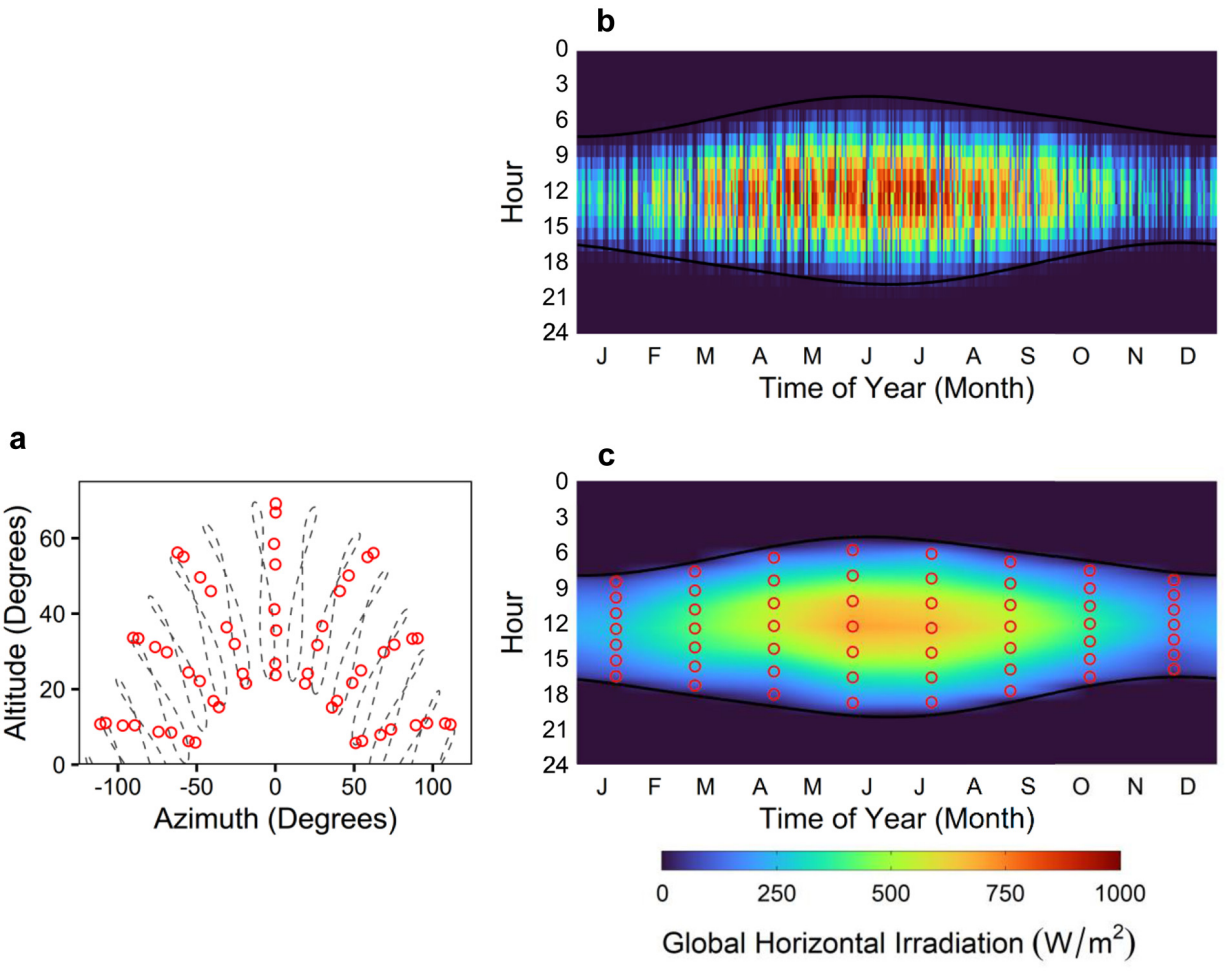
Time sampling strategy and climate information for Toronto, Canada: (a) shows the solar positions of the 56 sampled times according to solar altitude and azimuth overlaid on top of hourly local time analemmas; (b) shows the hourly global horizontal irradiance from the Canadian Weather for Energy Calculations (CWEC) climate data for Toronto^51^ and (c) illustrates the sampled times as points overlaid on top of an hourly interpolated map of global horizontal irradiation used by our implementation of the Lightsolve method

We extend ALFA’s physics-driven sky models to an annual simulation by implementing and automating the Lightsolve method.^50^ Fifty-six point-in-time simulations distributed throughout the year are calculated and interpolated to yield annual results – seven times per day for eight days during the year. The simulation times each day are evenly distributed between sunrise and sunset, while simulation days are evenly distributed between the first and last day of the year. Lighting conditions at these 56 times are based on the prevailing climatic conditions of Toronto and can be used to estimate typical light levels throughout the year.^51^ Figure 5 displays the sampling strategy: (a) indicates the sampled solar positions as red circles on top of hourly analemmas for the entire year in Toronto, (b) illustrates the hourly global horizontal irradiation from the Toronto climate data and (c) shows how irradiation is averaged and interpolated across the year based on the 56 sampled hourly times. Black lines in (b) and (c) indicate the time of sunrise and sunset.

We ran all the simulations using ALFA’s ‘hazy’ partially cloudy sky condition^29^ and scaled ALFA’s idealized spectral simulation conditions to match the mean global horizontal irradiation over the 56 Lightsolve method simulation intervals for Toronto. Pierson *et al*.^19^ found that this method of irradiance-based scaling produces simulated spectra with no bias when compared against measured data. This method misses peaks (completely clear sky days) and valleys (overcast days) of light exposure that would occur in reality, instead simulating typical sky conditions for the entire year. The hazy sky ranges in a range of CCTs between 5740 K and 6300 K throughout our 56 samples, whereas clear skies may have greater variation, especially in the very early sunrise and sunset times of day. The authors present these simulations as a way of evaluating typical lighting conditions as they change seasonally and throughout the day. They can be used to evaluate the general performance of a daylighting design and electric lighting control systems, but further analysis would be needed to explore performance under other specific weather conditions.

### 3.6 Light scenarios

To demonstrate the iNSOM simulation method, we apply a set of electric lighting and screen use scenarios combined with annual daylight access. The scenarios we apply are described below.

(1) *Daylight only (daylight)*– Light exposure is only due to daylight.
(2) *Daylight with constant electric light and monitor screen spectra (electric light)*– Daylight as per the daylight only scenario is present. Electric lighting with a CCT of 6500 K is turned on during all waking hours (6 am to midnight). Monitor screens with a blue-enriched screen with a CCT of 6500 K are used throughout the evening (6 pm to midnight).
(3) *Daylight, colour changing electric lighting and evening monitor screen spectra (dimming)*– Daylight is present as per scenarios 1 and 2. Electric light begins as per scenario 2 but shifts to a warm 2800 K spectrum from 6 pm to 10 pm in the evening and is dimmed to half power from 10 pm to midnight. The monitor screen spectra shifts to a blue-depleted 1900 K CCT from 9 pm to midnight to reduce ipRGC-influenced effects.

For all scenarios, a 10% transmissive, spectrally neutral roller blind is lowered when the photopic vertical eye illuminance of any of the 12 simulated views exceeds 3000 lx. The shade is raised whenever light levels would not exceed 3000 lx on any simulated view. In practice, for our East-facing simulation space, this means that shades are typically lowered during the morning hours and raised from early afternoon until the next day.

## 4. Results

### 4.1 Annual melanopic irradiance outputs

**Figure 6:**
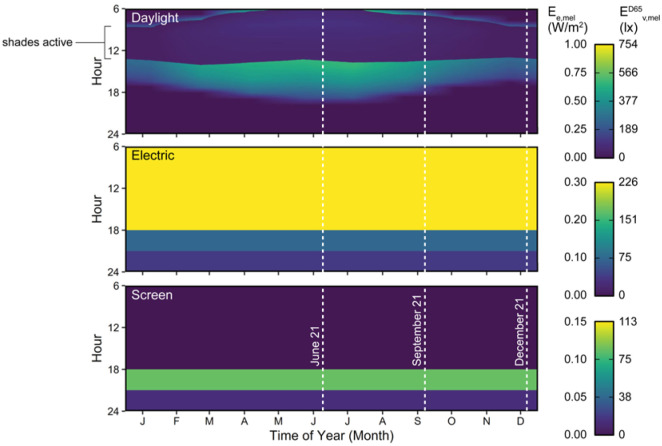
Melanopic irradiance experienced at the example view during scenario 3 from daylight, electric luminaires and screens over the year. Dashed white lines indicate daily data shown in Figure 7

**Figure 7:**
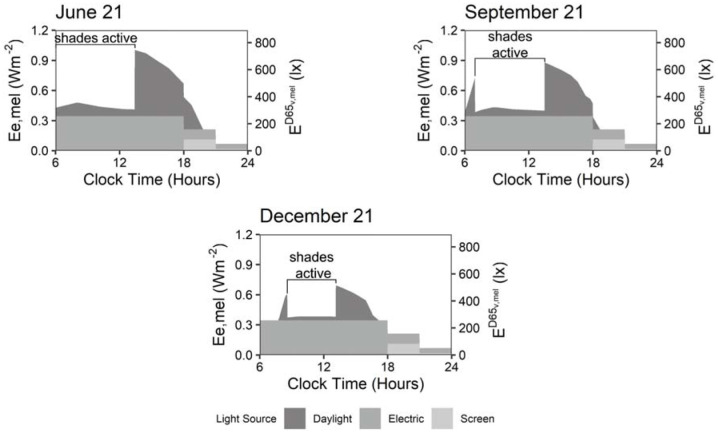
Melanopic irradiance experienced at the example view during scenario 3 from daylight, electric luminaires and screens over the year over the day in summer, fall and winter

Figures 6 and 7 illustrate the melanopic irradiance experienced at the example view from Figure 1 and under lighting scenario 3 (*daylight with white tuning luminaires and screen*). The three annual plots in Figure 6 demonstrate varying melanopic irradiance using a colour gradient over the months in a year on the *x*-axis and the hours in a day on the *y*-axis. The three daily plots in Figure 7 are sampled based on the vertical white lines on the annual plots. For the daily plots, colour (shade of grey) represents light source, the *y*-axis is the amount of melanopic irradiance and the *x*-axis is the hours in a day. The variation in daylight observable in Figure 6 demonstrates the utility of simulating time-varying light exposure. This variation is observable at the annual scale which is demonstrated in the top graph as higher levels of melanopic irradiance are received during the summer months than winter months during daylight hours and further demonstrated in the bottom three graphs as the peak melanopic irradiance is highest on 21st June and lowest on 21st December. This variation is also observable at the circadian or daily scale in the bottom three graphs as the melanopic irradiance due to daylight changes throughout the day depending on climate data and melanopic irradiance due to luminaires varies based on controls, either automatic or human-operated. Electric lighting and screen source exposures are mitigated by control systems which have a strong impact on ipRGC-influenced effects in non-daylit spaces during the day and in all spaces at night.

### 4.2 Mean melanopic irradiance outputs

**Figure 8:**
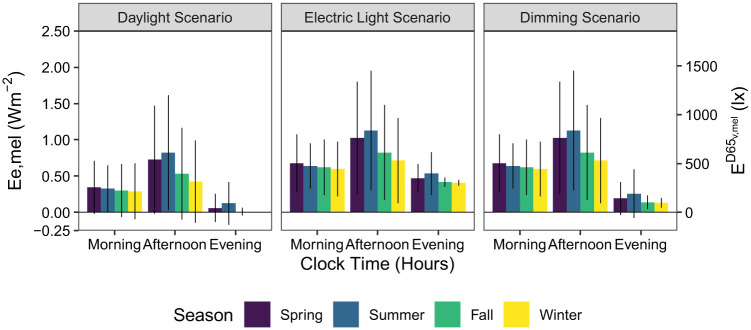
Mean melanopic irradiance experienced across all views for three lighting scenarios, four seasons and three times of day

Figure 8 illustrates the mean melanopic irradiance averaged across all 12 views indicated in Figure 1. Colours indicate the four seasonal temporal divisions: spring is purple; summer is blue; fall is green and winter is yellow. Vertical bars indicate the spread of ±1 SD across the 12 spatial results, indicating how much occupant position in the space influences the reception of melanopic irradiance at the eye. Results are further separated by the three lighting scenarios (major column organization) and three daily time periods (*x*-axis ticks): morning (8.00 to 12.00), afternoon (12.00 to 18.00) and evening (18.00 to 24.00). Quantitative visualizations such as this give potential lighting designers the ability to understand how much *E*_e,mel_ melanopic irradiance (W m^−2^) varies across a design and by time.

Mean melanopic irradiance is overall highest in the electric light scenario and lowest in the daylight scenario, as is expected. Furthermore, based on the error bars for the morning and evening daylit scenarios, daylight alone is insufficient to adequately light the simulated space, emphasizing the need for electric lighting. The difference in scenarios due to electric lighting is most notable during the evening period where the electric light scenario provides 2.5 times the dimming scenario and more than 9 times the daylight scenario (seasonal-spatial means over the evening time period of *E*_e,mel_ = 0.46 W m^−2^ ( $E_{v,\mathrm{mel}}^{D65}=344\;\mathrm{lx}$ ), *E*_e,mel_ = 0.18 W m^−2^ ( $E_{v,\mathrm{mel}}^{D65}=135\;\mathrm{lx}$ ) and *E*_e,mel_ = 0.05 W m^−2^ ( $E_{v,\mathrm{mel}}^{D65}=37\;\mathrm{lx}$ ), respectively). As a result, no scenarios meet the Brown *et al*.^40^ recommended threshold of $E_{v,\mathrm{mel}}^{D65}<10\;\mathrm{lx}$ before bedtime which suggests the need to further dim electric lighting and screen devices even beyond the targets set in the dimming scenario (blue-depleted spectral shift for electric lights and screen devices, 50% luminous intensity reduction for electric lights). In this paper, the evening period begins at 6 pm, while Brown *et al*.^40^ specify 3 h before bedtime.

Analysis of the daylight scenario explains lighting fluctuations due to daylight in the other two scenarios as well. For the daylight scenario, during the morning period when shades are mostly lowered, mean *E*_e,mel_ irradiances are greatest during spring (*E*_e,mel_ = 0.382 W m^−2^, $E_{v,\mathrm{mel}}^{D65}=288\;\mathrm{lx}$ ) and progressively slightly less through the remaining seasons by about 0.025 W m^−2^ per season – summer, fall and winter. During largely unshaded periods of time, afternoon daylight exposure significantly increases compared to the morning period –*E*_e,mel_ = 0.97 W m^−2^ ( $E_{v,\mathrm{mel}}^{D65}=728\;\mathrm{lx}$ ) during summer and *E*_e,mel_ = 0.50 W m^−2^ ( $E_{v,\mathrm{mel}}^{D65}=378\;\mathrm{lx}$ ) during winter. Daylight exposure is minimal during the evening period with melanopic irradiance as low as 0 W m^−2^ during winter.

### 4.3 Spatial melanopic irradiance outputs

**Figure 9:**
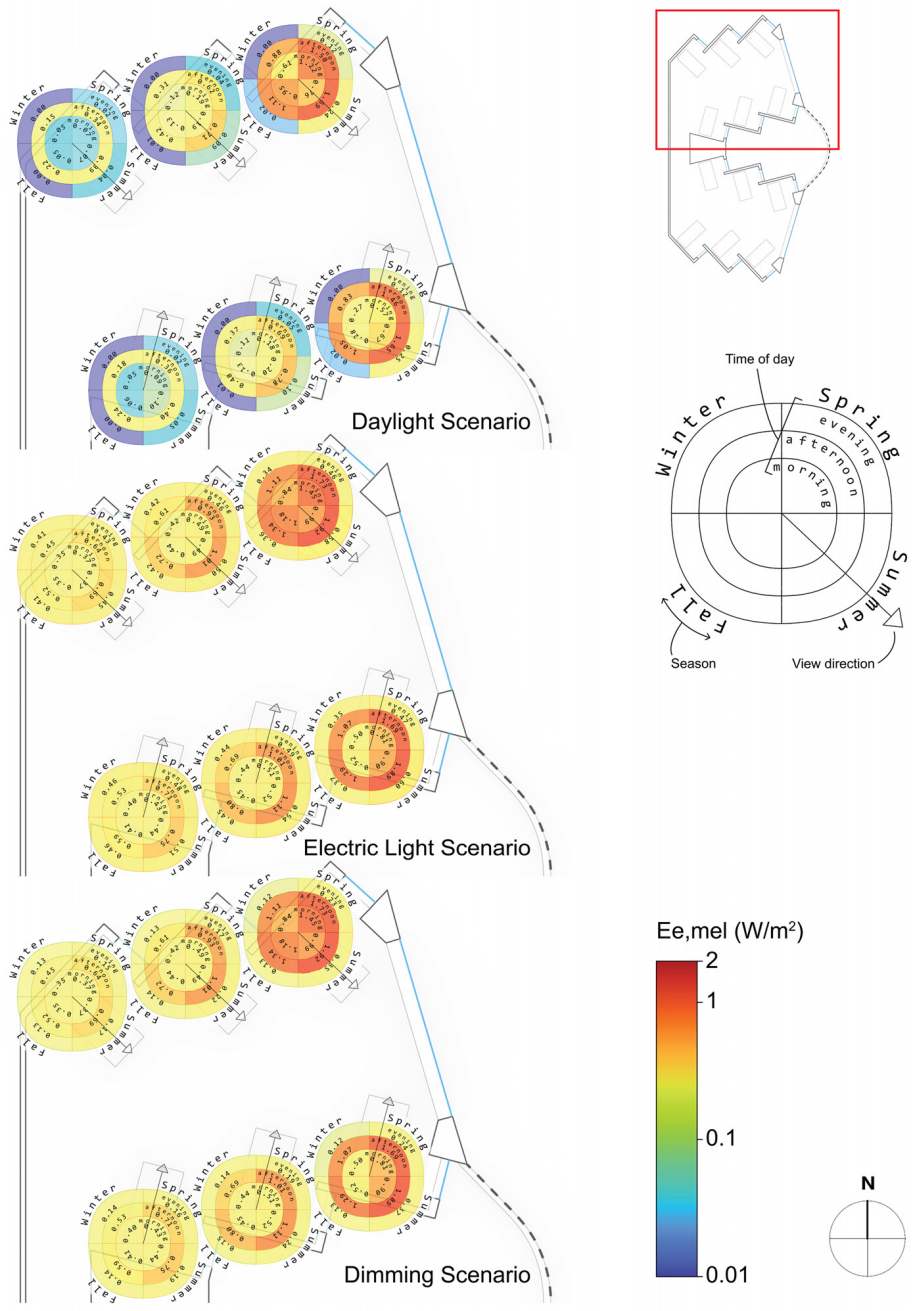
Spatially displayed melanopic irradiance by scenario, time of day and season

Spatial distribution of these results can illustrate scenario- and positional-differentiation in received melanopic irradiance within a well-daylit space. For conciseness, only the northern half of views within the hospital ward are shown in Figure 9 and discussed herein. Our visualization derives from a type of display called the ‘sombrero plot’, pioneered by Andersen, Mardaljevic and Roy.^14,43^ Their plot indicates multiple view directions by time of day whereas ours provides seasonal and time of day bins but only for a single view direction. While there is less spatial information presented in this manner, more seasonal and time-of-day variations can be illustrated for the most typical views using this display method. The middle-right legend of Figure 9 describes the style of the display – the arrow indicates view direction, quadrants of the oval diagram indicate season and concentric circles indicate time of day with morning being closest to the centre and evening in the outermost ring. A logarithmic colour scale is used (*E*_e,mel_ from 0.1 W m^−2^ to 2.0 W m^−2^) so that variation between scenarios can be visualized during bright daylit periods and during low evening light levels when electric light may be the primary contributor.

Across all three scenarios, morning period and afternoon irradiation varies the most spatially when daylight dominates. For example, the difference between the mean view melanopic irradiance at the easternmost front of the space and the back of the space during morning in the daylight scenario across all seasons is *E*_e,mel_ = (0.63 W m^−2^ − 0.09 W m^−2^) = 0.54 W m^−2^ or $E_{v,\mathrm{mel}}^{D65}=407\;\mathrm{lx}$ . However, during the darker evening, this difference is only *E*_e,mel_ = (0.09 W m^−2^ − 0.02 W m^2^) = 0.07 W m^−2^ or $E_{v,\mathrm{mel}}^{D65}=52\;\mathrm{lx}$ . Normalizing these differences by the average of all view irradiances during the same period, the difference between front and back during the morning is 159%, while during the evening it is 141%. The dimming scenario (and by extension the electric lighting scenario) exhibits relatively minimal spatial differentiation between the front and back during the same evening period –*E*_e,mel_ = (0.21 W m^−2^ − 0.15 W m^−2^) = 0.06 W m^−2^ or $E_{v,\mathrm{mel}}^{D65}=45\;\mathrm{lx}$ – 34% when normalized by the average view irradiance. In this case, the results suggest a relatively uniform distribution of electric light in the simulated model. Figure 9, by extension, is provided as a proof-of-concept visualization to assess the spatial and temporal results of the combined ipRGC-influenced lighting simulations from daylight, electric light and screen devices. In our follow-up paper, we will discuss the health and well-being implications of these results for theoretical patients in the hospital ward.

## 5. Discussion

The goal of this pair of papers is to bring together recent knowledge from building science on light simulation technology with the most recent medical knowledge on modelling the ipRCG-influenced system. This first paper proposes a method for producing annualized, climate-based and time-series melanopic irradiance simulations accounting for light from daylight, electric lighting and screens. However, this is a preliminary workflow, and there is more to do for the framework to model reality. Firstly, the paper assumes the occupant is completely stationary 24/7 which is unrealistic, and more information is needed on occupant habits throughout the day. Still, we believe that modelling a typical resting position and view provides useful information about lighting in the space.

Secondly, to the best of our knowledge, there currently is not a user interface or datasets available to model interactions between complex control systems for electric light and occupant behaviour like using blinds, movement within the building, occupant adaptation and screen use that will affect light exposure. Utilizing frameworks for modelling occupant light exposure throughout the day using typical occupant behaviour profiles, such as the work of Danell *et al*.^42^ is a promising future direction.

More work is also needed to validate Radiance 6 and ALFA’s skies using real-world measurements as the validation challenge for physics-based based skies derived under standard atmospheric profiles^20,23^ is a large one. ALFA has not yet been calibrated to use with standard ground-based weather data such as is common in photopic climate-based daylight simulation methods using the Perez sky model^25^ and ground-sensed or satellite-derived irradiation data. The method presented here does not need to be limited to ALFA as the Lightsolve method demonstrated in this paper can be applied to other multi-spectral tools too, and annual simulations using Lark 2.0 are promising. Radiance 6 has also extended the ‘N-phase’ methods to use annual spectral skies from Bruneton and Neyret. The ‘typical’, physics-based sky luminance and spectral distribution skies used in this paper are a first step in understanding seasonal spectral intensity variations in daylit spaces, and which part 2 of this paper discusses

## 6. Conclusion

Despite its limitations and required future work, iNSOM’s light calculation engine demonstrates a significant step forward for evaluating ipRGC-influenced lighting design. The new model simulates light in terms of its intensity, timing and spectrum which can be used to evaluate the CIE’s updated circadian light metrics, specifically those applied in photobiology. Throughout this paper, we use melanopic irradiance^48^ to describe these results; however, full spectral information is available and the entire suite of CIE S 026 toolkit metrics can be calculated. iNSOM incorporates light from three critical sources – daylight, electric light and screens – in the simulation of melanopic irradiance, which will be input to human photobiological performance models in the second part to this manuscript.
